# Insights and improvements in correspondence between axonal volume fraction measured with diffusion‐weighted MRI and electron microscopy

**DOI:** 10.1002/nbm.5070

**Published:** 2023-12-14

**Authors:** Sebastian Papazoglou, Mohammad Ashtarayeh, Jan Malte Oeschger, Martina F. Callaghan, Mark D. Does, Siawoosh Mohammadi

**Affiliations:** ^1^ Department of Systems Neuroscience University Medical Center Hamburg–Eppendorf Hamburg Germany; ^2^ Max Planck Research Group MR Physics Max Planck Institute for Human Development Berlin Germany; ^3^ Wellcome Centre for Human Neuroimaging, UCL Queen Square Institute of Neurology University College London London UK; ^4^ Department of Biomedical Engineering Vanderbilt University Nashville Tennessee USA; ^5^ Institute of Imaging Science Vanderbilt University Medical Center Nashville Tennessee USA; ^6^ Department of Radiology and Radiological Sciences Vanderbilt University Medical Center Nashville Tennessee USA; ^7^ Department of Electrical Engineering Vanderbilt University Nashville Tennessee USA; ^8^ Department of Neurophysics Max Planck Institute for Human Cognitive and Brain Sciences Leipzig Germany

**Keywords:** axonal volume fraction, axonal water fraction, biophysical model, calibration, diffusion‐weighted imaging, g ratio, histology reference, unmyelinated axons

## Abstract

Biophysical diffusion‐weighted imaging (DWI) models are increasingly used in neuroscience to estimate the axonal water fraction ( $f_{\text{AW}}$), which in turn is key for noninvasive estimation of the axonal volume fraction ( $f_{A}$). These models require thorough validation by comparison with a reference method, for example, electron microscopy (EM). While EM studies often neglect the unmyelinated axons and solely report the fraction of myelinated axons, in DWI both myelinated and unmyelinated axons contribute to the DWI signal. However, DWI models often include simplifications, for example, the neglect of differences in the compartmental relaxation times or fixed diffusivities, which in turn might affect the estimation of $f_{\text{AW}}$. We investigate whether linear calibration parameters (scaling and offset) can improve the comparability between EM‐ and DWI‐based metrics of $f_{A}$. To this end, we (a) used six DWI models based on the so‐called standard model of white matter (WM), including two models with fixed compartmental diffusivities (e.g., neurite orientation dispersion and density imaging, NODDI) and four models that fitted the compartmental diffusivities (e.g., white matter tract integrity, WMTI), and (b) used a multimodal data set including ex vivo diffusion DWI and EM data in mice with a broad dynamic range of fibre volume metrics. We demonstrated that the offset is associated with the volume fraction of unmyelinated axons and the scaling factor is associated with different compartmental $T_{2}$ and can substantially enhance the comparability between EM‐ and DWI‐based metrics of $f_{A}$. We found that DWI models that fitted compartmental diffusivities provided the most accurate estimates of the EM‐based $f_{A}$. Finally, we introduced a more efficient hybrid calibration approach, where only the offset is estimated but the scaling is fixed to a theoretically predicted value. Using this approach, a similar one‐to‐one correspondence to EM was achieved for WMTI. The method presented can pave the way for use of validated DWI‐based models in clinical research and neuroscience.

## Graphical abstract

We show for five biophysical diffusion weighted imaging (DWI) models, including WMTI and NODDI, that linear calibration with an offset ($U_2$) associated with pool of unmyelinated axons, and a scaling ($s$) associated with the different compartmental relaxation times, substantially improves the one‐to‐one correspondence between the axonal volume fraction from DWI and the volume fraction of myelinated axons from electron microscopy (EM). Furthermore, we show that the best one‐to‐one correspondence is achieved for DWI models fitting compartmental diffusivities.

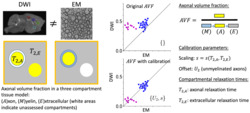

**Abbreviations**
ACID: artefact correction in diffusion MRI
ANOVA: analysis of variance
BAYDIFF: Bayesian estimation of microstructural diffusion parameters based on single and multiple MRI diffusion encodings
*BIC*
: Bayesian information criterion
CKO: conditional knockout
DWI: diffusion‐weighted imaging
EM: electron microscopy
Gd‐DTPA: gadolinium diethylenetriamine penta‐acetic acid
NODDI: neurite orientation dispersion and density imaging
PBS: phosphate‐buffered saline
Pten: phosphatase and tensin homolog
Rictor: rapamycin‐insensitive companion of mTOR
ROI: region of interest
*RSS*
: residual sum of squares
*SD*
: standard deviation
SMI: standard model imaging
Tsc2: tuberous sclerosis complex subunit 2
WM: white matter
WMTI: white matter tract integrity
WMTI‐W: WMTI–Watson

## 1 INTRODUCTION

Diffusion‐weighted imaging (DWI) is frequently used by neuroscientists as a noninvasive tool to infer microstructural tissue features. A range of biophysical multicompartment DWI models have been proposed to connect the diffusion‐weighted signal to the axonal water fraction,^1^, ^2^, ^3^, ^4^, ^5^ inspired by early biophysical models such as those of Assef et al. and Jespersen et al.^6^, ^7^ Most of these models are variants of the so‐called standard model of white matter.^8^ Such models are increasingly used in clinical research and neuroscience,^9^, ^10^, ^11^, ^12^, ^13^, ^14^ where the most widely employed DWI models are neurite orientation dispersion and density imaging (NODDI)^15^ and white matter tract integrity (WMTI).^16^ They have been employed, for example, for the estimation of the MR g ratio—a measure that is indicative of neuronal conduction velocity and thus of the functional integrity of white matter (WM) fibres.^4^, ^17^, ^18^

However, these biophysical DWI models include certain simplifying assumptions about the underlying tissue microstructure in order to meet the demand for reasonable measurement times and numerical stability of parameter estimation. One important limitation is that these models neglect different compartmental $T_{2}$ values in the intra‐ and extracellular signal.^19^, ^20^, ^21^, ^22^ The signals of the aforementioned multicompartment DWI models are modelled as the sum of signal contributions from the individual compartments (e.g. axonal, extracellular, isotropic compartments). The fraction of the signal of the axonal compartment is then usually directly related to the metric for the axonal water fraction by multiplication by a factor that accounts for the low sensitivity to the myelin water signal.^17^ Despite this correction factor, signal fraction and axonal water fraction are not truly interchangeable, because of the different transverse relaxation times ( $T_{2}$) in the compartments. Instead, the signal fractions are weighted fractions, the weights of which depend on the compartmental $R_{2}$ differences ( $R_{2}=1/T_{2}$) and the echo time $T_{E}$ employed.^19^, ^21^

Although challenges and limitations of these biophysical DWI models are well‐known,^3^ their accuracy has been investigated only to a limited extent. An analysis of the accuracy of DWI‐based axonal metrics requires an accurate reference. A frequently used method for measuring the axonal volume fraction is electron microscopy (EM), because its resolution allows one to distinguish between the myelin sheath and axonal body of single axons. While the fraction of unmyelinated axons can, in principle, also be assessed with EM,^23^, ^24^, ^25^ analyses typically focus solely on the fraction of myelinated axons, especially when performing EM on human brain tissue.^26^, ^27^, ^28^ This is because, compared with myelinated axons, unmyelinated axons are more difficult to distinguish from other entities such as, for example, glial cells, and hence more prone to misclassification than myelinated axons.^29^, ^30^, ^31^ On the other hand, DWI‐based estimates of the axonal volume fraction are not only sensitive to the volume fraction of myelinated axons but, presumably to a lesser degree, also affected by the unmyelinated axons.^32^ Therefore, testing the accuracy of DWI‐based estimates of axon volume fractions of myelinated axons by comparison with the EM reference would require a calibration step that corrects for potential differences in the sensitivity to unmyelinated axons and for potential limitations of DWI models.

In this study we demonstrate that linear calibration including an offset and a scaling can improve the comparability of DWI‐ and EM‐based axonal volume metrics and allows us to assess the accuracy of DWI‐based models of the volume fraction of myelinated axons. We hypothesise that the offset accounts for the differential sensitivity of our EM and DWI to the fraction of unmyelinated axons. Moreover, the linear calibration includes a scaling factor to account for compartmental $T_{2}$ differences. We compare different models with varying degrees of complexity. We investigate six DWI models based on the so‐called standard model of WM,^8^ including two models with fixed compartmental diffusivities, NODDI^15^ and NODDI–DTI,^33^ and four models that fitted the compartmental diffusivities. The latter included two relatively novel implementations, standard model imaging (SMI)^34^ and a Bayesian variant fitting the standard‐model parameters (BAYDIFF),^35^ as well as an older model (WMTI) ^16^ and a variant of it, WMTI–Watson (WMTI–W).^36^ For the comparison of DWI‐ and EM‐based models of the axonal volume fraction, we use a multimodal, ex vivo dataset of DWI and EM data of mouse WM from the corpus callosum and fornix.^37^, ^38^ Before comparing the DWI models with EM, we first perform a group selection based on mouse models using the EM‐based axon volume fraction as the selection criterion. Then, we determine the best combination of calibration parameters for each DWI model required to establish comparability with the EM reference. This combination of calibration parameters is also compared with a hybrid calibration approach, where the scaling calibration factor is determined analytically using a newly derived analytical approximation that relates the scaling parameter to the compartmental $T_{2}$ differences given $T_{E}$ while the offset parameter is estimated from the data. Finally, we assess the accuracy of the DWI‐based models of the volume fraction of myelinated axons achieved through the proposed, purely data‐driven and hybrid calibration approaches by comparison with their EM‐based counterpart.

## 2 BACKGROUND

### 2.1 DWI‐ and EM‐based metrics of axonal volume

**FIGURE 1:**
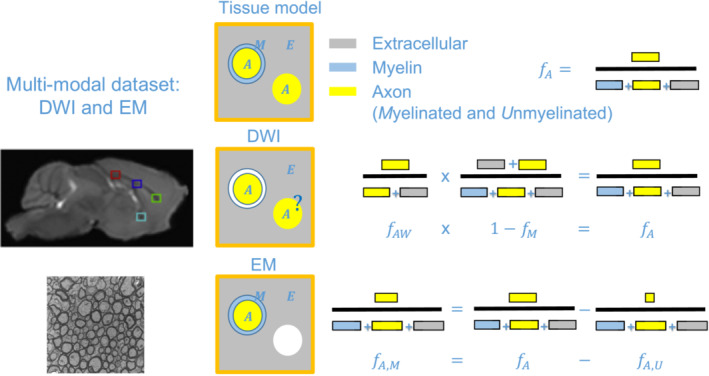
Schematic relating the tissue volume fractions of the three‐compartment tissue model to their counterpart from the multimodal dataset including DWI (top left) and EM (bottom left) data. Note that areas presented in white are not observable with the corresponding technique, that is, myelin in the case of DWI and unmyelinated axons in the case of EM. The question mark indicates that it is not known to what extent the fraction of unmyelinated axons can be estimated by the DWI models. DWI and EM images were taken from Kelm et al.^37^ and modified. Coloured boxes in the DWI image indicate regions of interest (ROIs) in which DWI metrics were available in this study (green: genu, blue: midbody, red: splenium, cyan: fornix).

White matter tissue is typically modelled as being composed of three distinct, nonoverlapping compartments quantified by the axonal ( $f_{A}$), myelin ( $f_{M}$), and extracellular volume fraction ( $f_{E}$), with

(1) $$f_{A}+f_{M}+f_{E}=1$$

in every WM voxel. A schematic description of the modelled volume fractions is shown in Figure 1. In DWI, in practice the myelin compartment is not affecting the signal, due to the short relaxation time of the myelin water. Therefore, the DWI signal is determined by the axonal water fraction $f_{\text{AW}}$. In the two‐compartment standard model of WM^8^ shown in the second row of Figure 1, $f_{\text{AW}}$ is given by

(2) $$f_{\text{AW}}=\frac{f_{A}}{f_{A}+f_{E}}.$$

Some DWI models also include an optional isotropic or CSF (cerebrospinal fluid) compartment ( $f_{\text{iso}}$) and for the ex vivo case an additional dot compartment ( $f_{\text{dot}}$) accounting for fixation effects, which would then have to be included in the sum on the left of Equation (1). In order to distinguish DWI‐based metrics from the “true” axonal water fraction $f_{\text{AW}}$ given by Equation (2), we denote them in the following by $f_{\text{AW}}^{\left(\text{DWI}\right)}\;(\approx f_{\text{AW}})$. To convert $f_{\text{AW}}$ (Equation 2) into the axonal volume fraction $f_{A}$, it has to be rescaled by $1-f_{M}$ (Figure 1, second row). We used electron microscopy metrics as the reference for both the axonal volume fraction of myelinated axons, denoted by $f_{A}^{\left(\text{EM}\right)}$, and the myelin volume fraction, denoted by $f_{M}^{\left(\text{EM}\right)}$. By rescaling $f_{\text{AW}}^{\left(\text{DWI}\right)}$ with the EM‐based prefactor $1-f_{M}^{\left(\text{EM}\right)}$, we obtain a DWI‐based estimate of the axonal volume fraction $f_{A}^{\left(\text{DWI}\right)}$:

(3) $$f_{A}^{\left(\text{DWI}\right)}=\left(1-f_{M}^{\left(\text{EM}\right)}\right)f_{\text{AW}}^{\left(\text{DWI}\right)}.$$

In practice, one would choose an MRI‐based metric for the myelin volume fraction to rescale the axonal water fraction. This typically requires additional calibration parameters to generate a volume fraction from the MRI‐based myelin marker. However, in this study we are interested in the effect of calibration parameters on the axonal volume fraction. Thus, to avoid ambiguity, we used the EM reference instead of an MRI‐based myelin marker as the metric for the myelin volume fraction. This ensures that any calibration is correcting for differences between EM and DWI due to the DWI‐based axonal water fraction only. Furthermore, we distinguish between myelinated ( $f_{\text{A,M}}$) and unmyelinated ( $f_{\text{A,U}}$) axon volume fractions (with $f_{A}=f_{\text{A,M}}+f_{\text{A,U}}$) to account for the fact that in this study only the first was assessed by EM, that is,

(4) $$f_{A}^{\left(\text{EM}\right)}=f_{\text{A,M}}.$$

A glossary of the metrics is given in Table 1.

### 2.2 Calibration parameters

In order to establish comparability between the EM reference $\left(f_{A}^{\left(\text{EM}\right)}\right)$ and the DWI‐based axonal volume fraction $\left(f_{A}^{\left(\text{DWI}\right)}\right)$, it is necessary to account for the differential sensitivity of the DWI models to the fraction of unmyelinated axons, which we model here by an additional offset calibration parameter U to $f_{A}^{\left(\text{DWI}\right)}$ (Equation 3):

(5) $$f_{A}^{\left(\text{DWI}\right)}\mapsto f_{A}^{\left(\text{DWI}\right)}-U\;(\text{offset calibration}).$$

Furthermore, in Equation (3) it is assumed that, apart from measurement error, $f_{\text{AW}}^{\left(\text{DWI}\right)}$ is equal to the true $f_{\text{AW}}$, which is a common assumption implying that compartmental $T_{2}$ differences in transverse relaxation are negligible. If compartmental differences cannot be neglected, the fraction of the axonal signal would become a function of the employed echo time $T_{E}$ with $f_{\text{AW}}^{\left(\text{DWI}\right)}(T_{E}=0)=f_{\text{AW}}$ (see, e.g., Gong et al.^21^). In the subsequent section it will be shown analytically that this $T_{E}$ dependence can be separated into a scaling calibration factor:

(6) $$f_{\text{AW}}^{\left(\text{DWI}\right)}\mapsto s(T_{E})f_{\text{AW}}^{\left(\text{DWI}\right)}\;(\text{scaling calibration}).$$

**TABLE 1:** Summary of the employed DWI and EM metrics and their relation with the tissue compartment model volume fractions.

Description	Tissue model metric	DWI metric	EM metric
Axonal volume fraction	$f_{A}$	$f_{A}^{\left(\text{DWI}\right)}$	–
Axonal volume fraction (unmyelinated)	$f_{\text{A,U}}$	–	–
Axonal volume fraction (myelinated)	$f_{\text{A,M}}$	–	$f_{A}^{\left(\text{EM}\right)}$
Axonal water fraction	$f_{\text{AW}}$	$f_{\text{AW}}^{\left(\text{DWI}\right)}$	–
Myelin volume fraction	$f_{M}$	–	$f_{M}^{\left(\text{EM}\right)}$
Extracellular volume fraction	$f_{E}$	–	–

### 2.3 Analytical derivation of the scaling calibration

All DWI models tested in this study (SMI, BAYDIFF, WMTI, WMTI–W, NODDI, and NODDI–DTI) are variants of the standard model.^8^ They can all be derived on the basis of the four‐compartment, ex vivo NODDI signal model, composed of axonal (with index a), extracellular, isotropic (iso), and so‐called dot (dot) compartments, the last of which accounts for water trapped inside small cavities in fixed tissue with effectively no diffusivity.^39^, ^40^ It is given by

(7) $$\frac{S_{\text{DWI}}}{S_{0}}=\left(1-\nu_{\text{dot}}\right)\left\{\left(1-\nu_{\text{iso}}\right)\left[\nu S_{A}+(1-\nu )S_{E}\right]+\nu_{\text{iso}}S_{\text{iso}}\right\}+\nu_{\text{dot}}S_{\text{dot}},$$

where $\nu_{\text{dot}},\;\nu_{\text{iso}}$, and ν are the signal fractions of the dot, isotropic, and axonal compartments, respectively. The usual in vivo NODDI model is obtained by setting $\nu_{\text{dot}}=0$ and the signal models for SMI, BAYDIFF, WMTI–W, WMTI, and NODDI–DTI are retrieved setting $\nu_{\text{dot}}=\nu_{\text{iso}}=0$. The compartmental signals ( $S_{\text{dot}},\;S_{\text{iso}}$, and $S_{a}$) are functions of the diffusion vector **b** and a set of biophysical parameters $\left\{p_{i}\right\}$, which depend on the DWI model (see Table 2), with the assumption that, at b=0, $S_{A}=S_{E}=S_{\text{iso}}=S_{\text{dot}}=1$. In analogy to the volume fractions defined in the tissue model shown in Figure 1, the signal fractions in Equation (7) are $\nu =f_{A}/(f_{A}+f_{E}),\;\nu_{\text{iso}}=f_{\text{iso}}/(f_{A}+f_{E}+f_{\text{iso}})$, and $\nu_{\text{dot}}=f_{\text{dot}}/(f_{A}+f_{E}+f_{\text{iso}}+f_{\text{dot}})$. In this signal model, the axonal water fraction is given directly by the corresponding coefficient of $S_{A}$: (ex vivo NODDI) $f_{\text{AW}}^{\left(\text{DWI}\right)}=(1-\nu_{\text{dot}})(1-\nu_{\text{iso}})\nu$, (in vivo NODDI and BAYDIFF, i.e., $\nu_{\text{dot}}=0$) $f_{\text{AW}}^{\left(\text{DWI}\right)}=(1-\nu_{\text{iso}})\nu$, and (SMI, WMTI–W, WMTI and NODDI–DTI, i.e., $\nu_{\text{dot}}=\nu_{\text{iso}}=0$) $f_{\text{AW}}^{\left(\text{DWI}\right)}=\nu$. This changes if the compartmental signals are functions of the corresponding compartmental transverse relaxation times. In that case $S_{0}$ becomes $S_{0}=\rho B_{1}^{+}B_{1}^{-}\hat{S_{0}}(t)$ ^41^ and $S_{\text{DWI}}$ changes correspondingly, where $B_{1}^{+}$ is the transmit profile, $B_{1}^{-}$ the receive profile, and ρ includes the proton density, which is assumed to be the same for all compartments. $\hat{S_{0}}(t)$ denotes the time‐dependent part of $S_{0}$ that remains after cancelling parts common with $S_{\text{DWI}}$ related to, for example, the head coil profile or proton density:

(8) $$\hat{S_{0}}(t)=\left(1-\nu_{\text{dot}}\right)\left\{\left(1-\nu_{\text{iso}}\right)\left[\nu e^{-T_{E}/T_{\text{2,a}}}+(1-\nu )e^{-T_{E}/T_{\text{2,e}}}\right]+\nu_{\text{iso}}e^{-T_{E}/T_{\text{2,iso}}}\right\}+\nu_{\text{dot}}e^{-T_{E}/T_{\text{2,dot}}}.$$

A simple signal model accounting for compartmental relaxation times is then given by

(9) $$\frac{S_{\text{DWI}}}{S_{0}}=\frac{1}{\hat{S_{0}}(t)}(\left(1-\nu_{\text{dot}}\right)\{\left(1-\nu_{\text{iso}}\right)\left[\nu S_{a}e^{-T_{E}/T_{\text{2,a}}}+(1-\nu )S_{e}e^{-T_{E}/T_{\text{2,e}}}\right]+\nu_{\text{iso}}S_{\text{iso}}e^{-T_{E}/T_{\text{2,iso}}}\}+\nu_{\text{dot}}e^{-T_{E}/T_{\text{2,dot}}}S_{\text{dot}}),$$

where $T_{E}$ is the echo time and $T_{\text{2,a}},\;T_{\text{2,e}},\;T_{\text{2,iso}}$, and $T_{\text{2,dot}}$ are the transverse relaxation times in the axonal, extracellular, isotropic, and dot compartments. For this signal model $S_{0}\neq 1$ and hence the coefficient of $S_{a}$ is now $f_{\text{AW}}^{\left(\text{DWI}\right)}(T_{E})=(1-\nu_{\text{dot}})(1-\nu_{\text{iso}})\nu e^{-T_{E}/T_{\text{2,a}}}/\hat{S_{0}}(t)$ and hence $T_{E}$‐dependent. The application of a model that does not account for compartmental relaxation to diffusion MRI data will therefore require a calibration scaling factor $s(T_{E})$ in order to retrieve the desired axonal water fraction from the coefficient of the axonal signal. As can be seen directly from the ex vivo NODDI signal model (Equation 9),

(10) $$s_{\text{pred}}\equiv \frac{\hat{S_{0}}(t)}{e^{-T_{E}/T_{\text{2,a}}}}=\left(1-\nu_{\text{dot}}\right)\left\{\left(1-\nu_{\text{iso}}\right)\left[\nu +(1-\nu )e^{-T_{E}\Delta_{e}}\right]+\nu_{\text{iso}}e^{-T_{E}\Delta_{\text{iso}}}\right\}+\nu_{\text{dot}}e^{-T_{E}\Delta_{\text{dot}}},$$

where $\Delta_{e}=1/(1/T_{\text{2,e}}-T_{\text{2,a}}),\;\Delta_{\text{iso}}=1/(1/T_{\text{2,iso}}-T_{\text{2,a}})$, and $\Delta_{\text{dot}}=1/(1/T_{\text{2,dot}}-T_{\text{2,a}})$. Then we have $s_{\text{pred}}\cdot f_{\text{AW}}^{\left(\text{DWI}\right)}=(1-\nu_{\text{dot}})(1-\nu_{\text{iso}})\nu \equiv f_{A}^{\left(\text{DWI}\right)}$ again. For the usual NODDI signal model $\nu_{\text{dot}}=0$ and for the remaining two‐compartment models of this study $\nu_{\text{dot}}=\nu_{\text{iso}}=0$. Hence the scaling can be predicted for the DWI models using Equation (10) once the compartmental signal fraction, compartmental $T_{2}$, and echo times are known from the literature or estimated from multi‐echo measurements such as described, for example, in Appendix B.

## 3 METHODS AND MATERIALS

We divided our analysis into three steps. First, we statistically assessed differences between the mouse models with respect to the EM‐based axonal volume fraction $f_{A}^{\left(\text{EM}\right)}$. In a second step, we determined, via data fitting, which combination of calibration parameters improves the one‐to‐one correspondence between the DWI‐based axonal volume fraction $f_{A}^{\left(\text{DWI}\right)}$ and its EM‐based counterpart $f_{A}^{\left(\text{EM}\right)}$ the most. Finally, we assessed the error and bias of the DWI models relative to the dynamic range in the EM data for the combinations found in the second analysis.

### 3.1 Dataset

The dataset used in this study is described in detail in Kelm et al. and West et al.^37^, ^38^ The data included DWI and EM histology data in an ex vivo cohort of N=15 mice. Six were healthy controls (i.e., $N_{\text{Controls}}=6$) and nine were genetically modified mouse models: three Pten CKO (hypermyelinated), three Rictor CKO (hypomyelinated), and three Tsc2 CKO (severely hypomyelinated), that is, ( $N_{\text{Pten}}=N_{\text{Rictor}}=N_{\text{Tsc2}}=3$). In total, for each mouse, diffusion kurtosis imaging (DKI) data (see, e.g., Kelm et al.^37^ for further details) and EM metrics $f_{A}^{\left(\text{EM}\right)}$ and $f_{M}^{\left(\text{EM}\right)}$ were available in the four aforementioned regions of interest (ROIs). This resulted in N=60 numerical values (15 mice × four ROIs) for each EM and DWI metric.

#### 3.1.1 Tissue preparation for DWI and EM

Tissue treatment for DWI and EM was as follows: in situ mouse brains were perfusion‐fixed using 2.5% glutaradehylde and 2% paraformadehylde + 1 mM Gd‐DTPA (Magnevist, Bayer HealthCare, Wayne, NJ, USA) in phosphate‐buffered saline (PBS). After excision, mouse brains were postfixed in the aforementioned fixative solution at 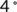
C for one week. After that the brains were washed throughly with PBS + 1 mM Gd‐DTPA at 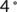
C for at least one week, with the solution being changed three times in order to wash out residual fixative that would reduce the tissue $T_{2}$.^42^

#### 3.1.2 DWI

For DWI imaging, the mouse brains were placed in MR‐compatible, perfluropolyether liquid‐filled tubes (Fomblin, Solvay Solexis, Thorofare, NJ, USA). Further DWI and DKI parameters were as follows: all DWI was performed on a 15.2T 11‐cm horizontal bore Bruker Biospec scanner (Bruker BioSpin, Billerica, MA, USA) at bore temperature ( 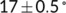
C), FOV = 19.2 x 14.4 x 10.8 mm^3^, matrix size = 128 x 96 x 72, at an isotropic resolution of 150 μm, that is, 22 500 $\mu m^{2}$ cross‐sectional voxel area. DKI was performed using a 3D diffusion‐weighted fast spin‐echo sequence. Further parameters were as follows: repetition time $T_{R}=200$ ms, echo time $T_{E}=19.0$ ms, gradient pulse duration δ=5 ms, diffusion time Δ=12 ms, b values = 3000 and 6000 s/mm^2^, 30 directions, and two signal averages with reversed gradient polarity.

#### 3.1.3 EM

After DWI, the brains were prepared for EM. To this end, thick midsagittal tissue sections were cut from the brains in four ROIs, three in the corpus callosum (genu, midbody, splenium) and one ROI in the fornix, as indicated by the coloured boxes in Figure 1. The sections were then placed in 1% osmium tetroxide in cacodylate buffer for one hour and dehydrated in graded ethanol. Then the tissue sections were embedded in epoxy resin and 1‐ μm thick sections were cut and stained with 1% toluidine blue.^37^ Finally, from the thick sections ROIs were selected using a standard mouse brain atlas and then ultrathin sections were cut for EM. EM‐based tissue metrics were assessed on images of size 2304 ×1888 pixels (controls) and 2048 ×1632 pixels (Pten, Rictor, and Tsc2) at a resolution of 0.004 ×0.009 $\mu m^{2}$, that is, with a total area of ≈156 $\mu m^{2}$ or ≈126 $\mu m^{2}$, respectively (see Vanderbilt University data at https://osf.io/yp4qg/).^43^ EM section size ranged between ≈10×10 and $40\times 40\;\mu m^{2}$, with 0.022μm thickness.

### 3.2 DWI model fitting

The DWI models were variants of the standard model of two nonexchanging compartments in which fibres are assumed to be impermeable sticks with no diffusion perpendicular to their orientation.^1^, ^8^ For a summary of the input parameters, biophysical (output) parameters, and assumptions of the DWI models, see Table 2. All DWI models except NODDI took as input all or a combination of the 21 standard DKI parameters, that is, the six independent elements of the diffusion tensor and 15 independent elements of the kurtosis tensor. The DKI parameters were estimated using the nonlinear least‐squares DKI framework implemented in the ACID toolbox (https://diffusiontools.com/, for further details see Appendix F).^44^ In each of the four ROIs, $f_{\text{AW}}^{\left(\text{DWI}\right)}$ was determined voxelwise. Overall, the number of voxels in the manually delineated ROIs ranged between six and 12. Then the mean value of $f_{\text{AW}}^{\left(\text{DWI}\right)}$ in the ROI was calculated, whereby voxels in which $f_{\text{AW}}^{\left(\text{DWI}\right)}<0,\;f_{\text{AW}}^{\left(\text{DWI}\right)}>1$, or $f_{\text{AW}}^{\left(\text{DWI}\right)}=\text{NaN}$ were discarded. This resulted in a reduced number of valid voxels only for a few DWI models, mouse individuals, and ROIs. All ROIs had at least four valid voxels, except for one which had only three valid voxels. Averaged over all ROIs and mouse individuals there were, per DWI model: BAYDIFF: 5% outliers (>1), WMTI–W^+^ 1% (NaN), and NODDI–DTI 5% (<0). SMI, WMTI, and NODDI had no outliers.

SMI: $f_{\text{AW}}^{\left(\text{DWI}\right)}$ was estimated using the standard model of WM as implemented and described at https://github.com/NYU-DiffusionMRI/SMI.^45^ In principle, this implementation of SMI allows modelling of two or three compartments including extra‐ and intracellular compartments and an isotropic compartment. Here, we chose the option to model only two compartments, which corresponds to discarding the isotropic compartment. To generate the noise map, we divided the signal at b=0 by the reported SNR=150.^37^ Furthermore, the machine‐learning bounds of the diffusion parameters were adjusted to fit the ex vivo situation of the mouse models (using the notation introduced in the present study): signal fraction of the axonal compartment ν∈(0.05,0.95), axonal diffusivity $D_{a}^{\|}\in (0.05,0.7)\;\mu m^{2}/\text{ms}$, extracellular parallel diffusivity $D_{e}^{\|}\in (0.05,0.7)\;\mu m^{2}/\text{ms}$, extracellular perpendicular diffusivity $D_{e}^{⊥}\in (0.05,0.7)\;\mu m^{2}/\text{ms}$, and free water (isotropic) compartment signal fraction $\nu_{\text{iso}}\in (0,0.5)$. No further parameters were fitted because of the single echo experiments used in this study.

BAYDIFF: $f_{\text{AW}}^{\left(\text{DWI}\right)}$ was estimated using the code from https://bitbucket.org/reisert/baydiff/wiki/Home.^35^ The prior distributions of the diffusivities used in the simulations for the initial training were adjusted to fit the ex vivo situation: all intra‐ and extra‐axonal diffusivities were assumed to be uniformly distributed in the interval $(0.05,0.7)\;\mu m^{2}/\text{ms}$. The same noise map as for SMI was used.

WMTI: $f_{\text{AW}}^{\left(\text{DWI}\right)}$ was estimated using the WMTI model^16^ implemented at https://github.com/NYU-DiffusionMRI/DESIGNER. WMTI has four free parameters, two extracellular diffusivities, one parallel and one perpendicular to the fibres, one intracellular diffusivity parallel to the WM fibres, and the axonal water fraction. Fibres are assumed to be parallel.

WMTI–W: $f_{\text{AW}}^{\left(\text{DWI}\right)}$ was estimated using an in‐house fitting algorithm implementation of the biophysical model introduced by Jespersen et al.^36^ The model has five free parameters, two extracellular diffusivities, one parallel and one perpendicular to the fibres, one intracellular diffusivity parallel to the WM fibres, the dispersion of fibres, and the axonal water fraction. Furthermore, due to the degeneracy of its solution, WMTI–W possesses two branches WMTI–W^+^ and WMTI–W^−^. The two branches include different assumptions on the compartmental diffusivities parallel to the direction of fibres: $D_{a}^{\|}>D_{e}^{\|}$ (WMTI–W^+^) and $D_{a}^{\|}<D_{e}^{\|}$ (WMTI–W^−^). Since the negative branch is known to yield unphysical results related to the absence of a proper minimum in its objective function^36^ and because, for in vivo application, the plus branch has been shown to be preferable,^46^ we discarded the negative branch from our analyses.

NODDI: $f_{\text{AW}}^{\left(\text{DWI}\right)}$ was estimated from the ex vivo NODDI model^15^ implemented in the NODDI MATLAB toolbox (https://www.nitrc.org/projects/noddi_toolbox). NODDI is the only three‐compartment (four compartments in ex vivo) model. In addition to the extracellular and axonal compartments that are shared by all DWI models of this study, it includes isotropic and cerebrospinal fluid compartments (and a dot compartment for the ex vivo case). The free parameters of in vivo NODDI are fibre dispersion, axonal water fraction, and isotropic water fraction and the ex vivo NODDI model features an additional signal fraction of the dot compartment (restricted water pool^39^, ^40^). The diffusivities for the isotropic compartment ( $D_{\text{iso}}$) and extracellular compartment, parallel to the fibre direction ( $D_{e}^{\|}$), were set to 2 and 0.35 $\mu m^{2}$/ms, respectively, as proposed in West et al.^38^

NODDI–DTI: $f_{\text{AW}}^{\left(\text{DWI}\right)}$ was determined from the aforementioned DKI fit using the fractional anisotropy ( FA) and mean diffusivity ( MD) from the standard DKI model as input. The FA and MD maps used as input for NODDI–DTI were calculated from the DKI fit as recommended in Edwards et al.^33^ to avoid a kurtosis bias in MD. NODDI–DTI features only two free parameters, fibre dispersion and axonal water fraction. The compartmental diffusivities are fixed as with NODDI.

**TABLE 2:** Summary by DWI model of the input data, the free biophysical parameters $p_{j}$, and the assumptions on them for the four validated DWI models. Symbols are as follows: ( $D_{\|}$) parallel diffusivity, ( $D_{⊥}$) perpendicular diffusivity, ( $W_{\|}$) parallel kurtosis, ( $W_{⊥}$) perpendicular kurtosis, ( ⟨W⟩) mean kurtosis, ( FA) fractional anisotropy, ( MD) mean diffusivity, ( $D_{e}^{\|},\;D_{e}^{⊥}$) diffusivities in the extracellular compartment, ( $D_{a}^{\|}$) diffusivity in the axonal compartment, ( κ) fibre dispersion, ( ν) axonal signal fraction, ( $\nu_{\text{iso}}$) and ( $\nu_{\text{dot}}$) signal fractions of the isotropic and dot compartments, respectively. Finally, $p_{2}$ is a rotational invariant of the fibre orientiation distribution function and represents an anisotropy metric.^45^

DWI model	Input	Biophysical parameters $p_{j}$	Assumptions
SMI	DWI data, noise map	$D_{e}^{\|},\;D_{e}^{⊥},\;D_{a}^{\|},\;\nu ,\;p_{2}$	$\nu_{\text{dot}}=\nu_{\text{iso}}=0$
BAYDIFF	DWI data, noise map	$D_{e}^{\|},\;D_{e}^{⊥},\;D_{a}^{\|},\;\nu ,\;\nu_{\text{iso}}$	$\nu_{\text{dot}}=0$
WMTI–W^+^	$D_{\|},\;D_{⊥},\;\langle W\rangle ,\;W_{\|},\;W_{⊥}$	$D_{e}^{\|},\;D_{e}^{⊥},\;D_{a}^{\|},\;\kappa ,\;\nu$	$\nu_{\text{dot}}=\nu_{\text{iso}}=0$
			$D_{a}^{\|}>D_{e}^{\|}$
WMTI	All 21 DKI parameters	$D_{e}^{\|},\;D_{e}^{⊥},\;D_{a}^{\|},\;\nu$	$\nu_{\text{dot}}=\nu_{\text{iso}}=0,\kappa \to \infty$
NODDI	DWI data	in vivo: $\kappa ,\;\nu ,\;\nu_{\text{iso}}$	$D_{e}^{\|}=D_{a}^{\|}$
		ex vivo: $\kappa ,\;\nu ,\;\nu_{\text{iso}},\;\nu_{\text{dot}}$	$D_{e}^{⊥}=(1-\nu )D_{e}^{\|}$
			$D_{\text{iso}}=2.0\;\mu m^{2}$/ms
			$D_{e}^{\|}=0.35\;\mu m^{2}$/ms
NODDI–DTI	FA,MD	κ,ν	$\nu_{\text{dot}}=\nu_{\text{iso}}=0$
			$D_{e}^{\|}=D_{a}^{\|}$
			$D_{e}^{⊥}=(1-\nu )D_{e}^{\|}$
			$D_{e}^{\|}=0.35\;\mu m^{2}$/ms

### 3.3 Statistical group selection

To prevent calibration parameter fitting from modelling noisy data, we assessed differences in $f_{A}^{\left(\text{EM}\right)}$ across the mouse models in terms of analysis of variance (ANOVA) with the null hypothesis that the mean value of $f_{A}^{\left(\text{EM}\right)}$ was the same across all models.

### 3.4 Best calibration parameter combinations

#### 3.4.1 Calibration parameter combinations

The combinations of linear calibration parameters that could potentially improve the one‐to‐one correspondence between DWI and EM were determined as follows: the case without any additional parameters corresponding to the assumption that $f_{A}^{\left(\text{DWI}\right)}\equiv f_{A}$ as given in Equation (3) defined the baseline. We then pooled the 15 individual mice and four ROIs into two groups according to the results from an ANOVA. Group 1 included healthy controls and only moderately hyper‐ or hypomyelinated mice (Pten or Rictor mouse models) and group 2 only included heavily hypomyelinated mice (Tsc2 mouse model), respectively. This choice was based on our finding that only between these two groups could a significant difference in the EM‐based axonal volume fraction $f_{A}^{\left(\text{EM}\right)}$ be observed, and not between any of the mouse models in the first group. Then we allowed for the estimation of individual offsets in each of the two groups (1: Controls, Pten, Rictor, and 2: Tsc2). For the purpose of optimisation they were written as column vectors $U_{j}=U_{j}e_{j}$ (with $U_{j}$ being the offset of group j and $e_{j}$ being a $N_{j}\times 1$ vector of ones) with $j\in \{1,2\},\;N_{1}=48$, and $N_{2}=12$ (for all DWI models). In this notation, Equation (3) including an offset calibration (Equation 5) and scalar scaling calibration (Equation 6) reads

(11) $$f_{A}^{\left(\text{DWI}\right)}=\left(1-f_{M}^{\left(\text{EM}\right)}\right)\cdot s\cdot f_{\text{AW}}^{\left(\text{DWI}\right)}-U,$$

where $U={\left[U_{1}^{T},U_{2}^{T}\right]}^{T}\in (N_{1}+N_{2},1)$, $f_{M}^{\left(\text{EM}\right)}$ and $f_{\text{AW}}^{\left(\text{DWI}\right)}$ indicate column vectors with components sorted in agreement with U, and s is the scaling calibration parameter. We considered the following combinations of calibration parameters: $U_{1}=0$ and $U_{2}\neq 0$ or $U_{1}\neq 0$ and $U_{2}=0$, and also both offsets were allowed to vary individually at the same time, that is, $U_{1}\neq U_{2}\neq 0$. In total, for each DWI model, we analysed seven combinations of the calibration parameters, denoted in the following as $\left\{U_{1}\},\;\{U_{2}\},\;\{s\},\;\{U_{1},U_{2}\},\;\{U_{1},s\},\;\{U_{2},s\},\;\{U_{1},U_{2},s\right\}$, and the baseline {}. For a summary of the calibration parameters tested, see also Table 3.

**TABLE 3:** Summary of the tested calibration parameters. Groups of mice are defined in Figure 2.

Description	Calibration parameter
Estimate of $f_{\text{A,U}}$ of the first group of mice	$U_{1}$
Estimate of $f_{\text{A,U}}$ of the second group of mice	$U_{2}$
Scaling accounting for differences in compartmental $T_{2}$	s

#### 3.4.2 Data‐driven calibration parameter estimation

The offsets $U_{j}$ and the scaling parameter s were estimated by minimising the residual sum of squares ( RSS) between the DWI‐based estimate for the axonal volume (Equation 11) and the EM‐based gold standard (defined in Equation 4):

(12) $$RSS={\left(f_{A}^{\left(\text{EM}\right)}-f_{A}^{\left(\text{DWI}\right)}\right)}^{T}\cdot \left(f_{A}^{\left(\text{EM}\right)}-f_{A}^{\left(\text{DWI}\right)}\right),$$

where again the bold‐faced quantities represent vectors including all available numerical values assembled into column vectors. In order to ensure physically reasonable estimates of the axon volume fraction, the optimization function (Equation 12) had to be complemented by a boundary condition. The constraint concerns the upper limit of the sum of volume fractions, that is, $\min\left(f_{A}^{\left(\text{DWI}\right)}+f_{M}^{\left(\text{EM}\right)}\right)-1<0$. Further constraints were lower and upper bounds for the calibration parameters: $U_{j}\in [0,1]$ and s∈[0,2]. All parameter estimations were performed using the nonlinear equation solver fmincon as implemented in Matlab 2020a (Mathworks, CA, USA).

To quantify the intramodel performance improvement of each DWI model due to the calibration parameters $U_{j}$ and s, we used the Bayesian information criterion ( BIC)^47^:

(13) $$BIC=k\ln n+n\ln\frac{RSS}{n},$$

where k is the number of model parameters, which varied between zero (baseline {}) and three depending on the combination of $U_{j},\;n$ is the number of evaluated data points, and RSS is defined in Equation (12). The BIC measures a model's capability of explaining given data while penalising overfitting. A lower BIC indicates less information loss, meaning that the model with the lowest BIC explains the data best. Since we employed the uncalibrated case {} as baseline, we only report differences ΔBIC with respect to this case, that is,

(14) $$\Delta BIC=BIC-BIC_{\left\{\right\}}.$$

ΔBIC was always calculated using all available data. For assessing the variation in $U_{j}$ and s, we performed a leave‐one‐out analysis by successively discarding the data of one mouse until each mouse was excluded once.

#### 3.4.3 Hybrid calibration parameter estimation

To simplify the demand on the distribution of the calibration data, we introduced a hybrid calibration approach. To this end, we used the best calibration parameter combination determined by the data‐driven approach described in Section 3.4.2 and estimated the offset calibration parameter $U_{j}$, while fixing the scaling parameter. In this approach, the scaling parameter was fixed to the theoretically predicted value (Equation 10) using compartmental $T_{2}$ estimates derived in Appendix B and only the offset $U_{j}$ was estimated using Equation (12).

### 3.5 Assessment of bias and error

For comparison of the accuracy achieved by the data‐driven (Section 3.4.2) and hybrid calibration (Section 3.4.3) approaches, we performed a Bland–Altman (BA) analysis^48^ of the differences

(15) $$\delta =f_{A}^{\left(\text{DWI}\right)}-f_{A}^{\left(\text{EM}\right)}$$

versus the mean

(16) $$m=\frac{1}{2}\left(f_{A}^{\left(\text{DWI}\right)}+f_{A}^{\left(\text{EM}\right)}\right).$$

The error was given by

(17) $$\epsilon =1.96\sqrt{\langle \delta^{2}\rangle -{\left\langle \delta \right\rangle }^{2}}.$$

We also report the mean difference ⟨δ⟩, that is, the bias, and bias $\bar{\delta }$ and error $\bar{\epsilon }$ relative to the dynamic range in the EM‐based axonal metric, that is,

(18) $$\bar{\delta }=\frac{\left\langle \delta \right\rangle }{\Delta f_{A}^{\left(\text{EM}\right)}}$$

and

(19) $$\bar{\epsilon }=\frac{\epsilon }{\Delta f_{A}^{\left(\text{EM}\right)}},$$

where $\Delta f_{A}^{\left(\text{EM}\right)}=f_{\text{A,max}}^{\left(\text{EM}\right)}-f_{\text{A,min}}^{\left(\text{EM}\right)}$ and angled brackets indicate an average.

## 4 RESULTS

### 4.1 Statistical group selection

**FIGURE 2:**
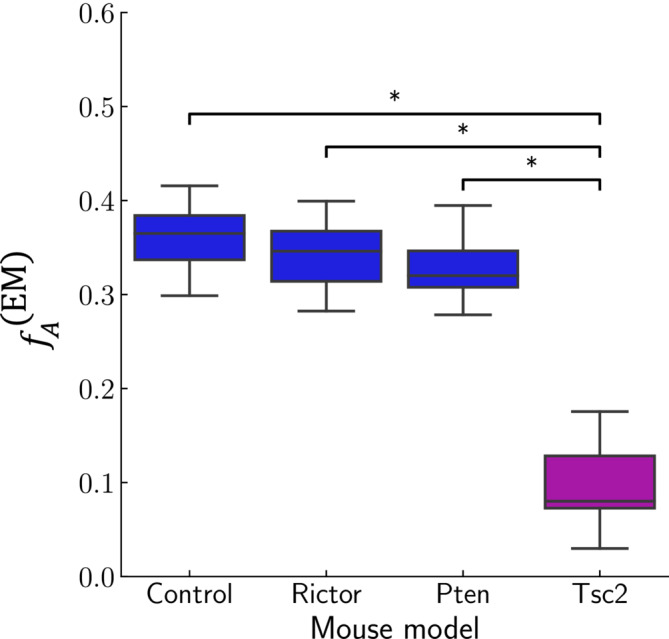
EM‐based axonal volume fraction $f_{A}^{\left(\text{EM}\right)}$ for the four mouse models: Controls, Pten, Rictor, and Tsc2. An ANOVA revealed significant differences ( p<0.05) only between Tsc2 and Rictor, Controls, or Pten, respectively. No further significant differences were observed. This motivated the pooling of the data into two groups: (1) Controls, Rictor, and Pten, and (2) Tsc2.

The result of the ANOVA analysis (Section 3.3) shown in Figure 2 revealed a significant ( p<0.05) difference between the EM‐based axonal volume fraction $\left(f_{A}^{\left(\text{EM}\right)}\right)$ of Tsc2 mice and any of the other models, while no significant differences were observed among Pten, Rictor, and Controls. As a consequence, the four mouse models were pooled into two groups: (1) Controls, Pten, Rictor, and (2) Tsc2 for further analysis.

### 4.2 Best calibration parameter combinations

#### 4.2.1 Data‐driven calibration parameter estimation

**FIGURE 3:**
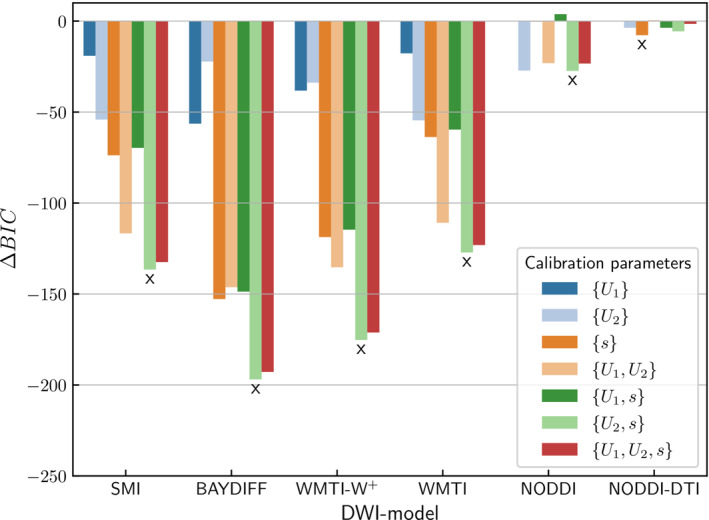
Contribution of calibration parameters to DWI model improvement. Shown are the differences ΔBIC (Equation 14) with respect to the parameter combination with the smallest BIC in each DWI model. A lower value indicates better model performance. Values for the uncalibrated case {} served as baseline, that is, for this case ΔBIC=0. An x indicates the calibration parameter combination with the largest evidence of improvement with respect to the baseline without further calibration for each DWI model, respectively.

In order to determine the best combination of calibration parameters, we performed a BIC (Equation 13) analysis. ΔBIC (Equation 14) for the tested parameter combinations are shown in Figure 3 for each DWI model separately. For all DWI models except NODDI–DTI, greatest evidence for improvement was achieved for the $\left\{U_{2},s\right\}$ set of calibration parameters, that is, when the offset for the severely hypomyelinated group 2 ( $U_{2}$) was combined with the scaling ( s). For NODDI–DTI, {s} had the lowest ΔBIC. Table 4 summarises the offsets $U_{2}$ and scaling s for the best combination of calibration parameters as indicated in Figure 3. The estimated offset $U_{2}$ for the Tsc2 mouse model varied between 0.18 and 0.24 for SMI, BAYDIFF, WMTI, and WMTI–W^+^. The scaling varied between 0.52 and 1.11 (note that scaling s=1 is equivalent to no additional scaling calibration). For a summary of all tested calibration parameter combinations see Table A1 in the Appendix.

**TABLE 4:** Summary of volume fraction of unmyelinated axons $U_{2}$, scaling s, and relative difference between fit s and theory $s_{\text{pred}}$, that is, $\Delta s=(s-s_{\text{pred}})/s_{\text{pred}}$, for the best parameter combinations from the first analysis (see also Figure 3). $U_{2}$ and s were estimated in a leave‐one‐out fashion, in which each mouse individual was excluded from the computation once in order to get an estimate of the standard deviation (see also the final paragraph in Section 3.4.2). Note that a value of 0 corresponds to exactly zero, while 0.0 corresponds to <0.005.

DWI model	Calibr. parameters	$U_{2}$ ( SD)	s ( SD)	$s_{\text{pred}}$	Rel. Δs [%]
SMI	$\left\{U_{2},s\right\}$	0.24 (0.01)	0.72 (0.01)	0.89	−19.0
BAYDIFF	$\left\{U_{2},s\right\}$	0.18 (0.0)	0.52 (0.0)	0.93	−44.0
WMTI–W^+^	$\left\{U_{2},s\right\}$	0.21 (0.0)	0.59 (0.01)	0.89	−33.0
WMTI	$\left\{U_{2},s\right\}$	0.21 (0.0)	0.76 (0.01)	0.89	−14.0
NODDI	$\left\{U_{2},s\right\}$	0.21 (0.01)	1.11 (0.01)	0.9	22.0
NODDI–DTI	{s}	0	0.87 (0.02)	0.89	−2.0

#### 4.2.2 Estimation of the theoretically predicted scaling calibration $s_{\text{pred}}$

Using the derived expression for the scaling calibration in Equation (10) together with the rescaled compartmental $T_{2}$ times (Equations B2), $T_{E}=19$ ms, and the mean ν=0.475 from Veraart et al.^19^ and Gong et al^21^ and $\nu_{\text{iso}}=0.05$,^21^ we found $s_{\text{pred}}\approx 0.93$ for BAYDIFF, $s_{\text{pred}}\approx 0.9$ for NODDI, and $s_{\text{pred}}\approx 0.89$ for the other DWI models. Table 4 shows that the smallest relative difference between fitted and predicted scaling Δs was found for NODDI–DTI (−2%) and the largest relative difference was found for BAYDIFF (−44%).

### 4.3 Bias and error of the best parameter combinations

**FIGURE 4:**
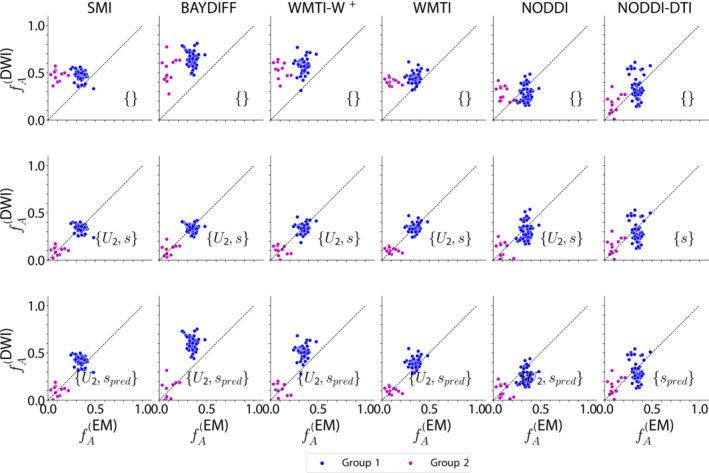
Scatter plots showing gold standard $f_{A}^{\left(\text{EM}\right)}$ (EM) versus DWI‐based estimates of the axonal volume fraction ( $f_{A}^{\left(\text{DWI}\right)}$). The first row shows the baseline, that is, without additional calibration parameters, and subsequent rows show the best (in terms of ΔBIC) calibration parameter combinations (see also Figure 3 and Table 4) with both parameters determined based on data (second row) and with the scaling fixed to the predicted values (last row). Data shown pool four ROIs per mouse individual. The data were divided into two groups determined by statistical distinguishability observed in the EM gold standard $f_{A}^{\left(\text{EM}\right)}$ (see Figure 2). The two groups are: (1) controls, Rictor, and Pten (blue), and (2) Tsc2 (magenta). Note that a corresponding comparison of the DWI‐based axonal water fraction $f_{\text{AW}}^{\left(\text{DWI}\right)}$ with the EM‐based axonal volume fraction $f_{A}^{\left(\text{EM}\right)}$ is shown in Figure C1 in the Appendix.

In Figure 4 we compare scatter plots of the histological reference $f_{A}^{\left(\text{EM}\right)}$ versus its DWI‐based counterpart $f_{A}^{\left(\text{DWI}\right)}$. The first row of the figure shows the baseline (i). SMI, BAYDIFF, WMTI–W^+^, and WMTI clearly overestimated the axonal volume fraction of myelinated axons in both groups of mice, indicated by the global offset from the line of unity. For NODDI, only the Tsc2 mice featured an obvious positive offset, while for NODDI–DTI all mouse models showed considerably better one‐to‐one correspondence, although with large variance along $f_{A}^{\left(\text{DWI}\right)}$. The second row shows the best calibration parameter combinations (ii). A substantially improved one‐to‐one correspondence was observed only for SMI, BAYIDFF, WMTI–W^+^, and WMTI, while NODDI and NODDI–DTI only improved a little or not visibly at all. The third row shows the scatter plots for the case in which the scaling was fixed to its predicted value and the offset was determined by fitting to the data (iii). There, the one‐to‐one correspondence is similar to the second row (ii) only for SMI and WMTI, while all other DWI models except NODDI–DTI show a less good one‐to‐one correspondence. Interestingly, it was mainly the control group for which the correspondence achieved between DWI and EM was less good than in case (ii). Again, NODDI–DTI displayed no visible changes compared with either (i) or (ii).

**FIGURE 5:**
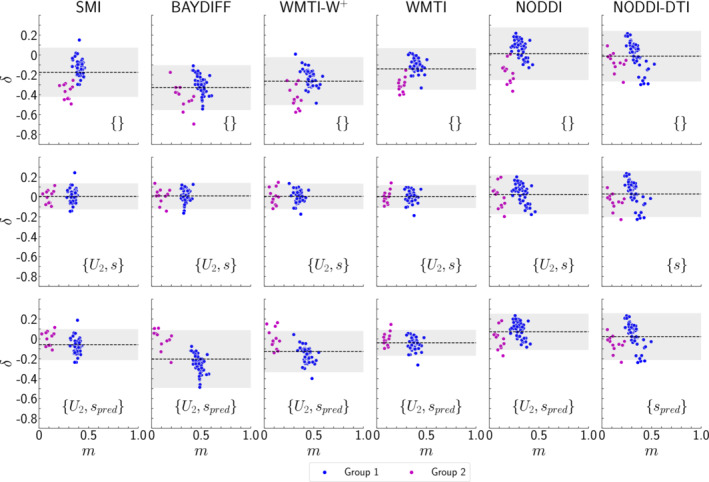
Bland–Altman plots of differences δ (Equation 15) versus mean m (Equation 16) between EM and DWI for the baseline (first row) and the best‐performing calibration parameter combination, where all parameters were estimated on the basis of data (second row) and the scaling was fixed to the theoretically predicted values (last row). The dashed line corresponds to the bias ⟨δ⟩, while the shaded region corresponds to ⟨δ⟩±ϵ (see Section 3.5). Individual data shown are in correspondence with Figure 4. Note that a corresponding comparison of the DWI‐based axonal water fraction $f_{\text{AW}}^{\left(\text{DWI}\right)}$ with the EM‐based axonal volume fraction $f_{A}^{\left(\text{EM}\right)})$ is shown in Figure C2 in the Appendix.

The capability to predict the EM‐based reference is quantified in terms of BA plots, shown in Figure 5, and bias and error relative to the dynamic range of the EM reference, summarised in Table 5. The results for the same calibration parameter combinations (i)–(iii) as in Figure 4 are shown. The BA plots show a substantial reduction in bias and error for the best calibration parameter combinations only for SMI, BAYDIFF, WMTI–W^+^, and WMTI. For NODDI, only the error was reduced, and for NODDI–DTI no improvement was observed at all.

Error and bias relative to the dynamic range in the reference $f_{A}^{\left(\text{EM}\right)}$ were substantially reduced for the best combination of calibration parameters (ii) only for SMI, BAYDIFF, WMTI–W^+^, and WMTI, whereby the relative bias was close to zero after calibration (see Table 5). BAYIDFF benefited the most in terms of relative bias, showing a reduction of 75% (from −73% to 2%). The largest reduction of relative error was observed for SMI with 26% (from 55% to 29%). NODDI and NODDI–DTI benefited much less from calibration. Their relative errors could be improved by 15% (NODDI) and 5% (NODDI–DTI). Their relative biases, however, increased slightly by 3% or 10%, respectively. Overall, the lowest relative error after calibration of all DWI models was observed for WMTI (26%). When the scaling was fixed to the theoretically predicted values and only the offset $U_{2}$ was determined on the basis of data (iii), similar improvement of the relative error to before, that is, for (ii), could only be achieved for SMI and WMTI. Improvement of the relative bias was less than in the case of the purely data‐driven approach (ii).

**TABLE 5:** Summary of the metrics assessed to validate the capability of the recalibrated DWI models to predict the EM‐based gold standard. Shown are bias (relative bias, Equation 17) and error (relative error, Equation 18) for the baseline and the best‐performing single‐ and multiparameter combinations. Corresponding values for the comparison of EM‐based axonal volume fraction $f_{A}^{\left(\text{EM}\right)})$ and DWI‐based axonal water fraction $f_{\text{AW}}^{\left(\text{DWI}\right)}$ are given in Appendix Table C1. Note that in Table D1 we provide an alternative version of this table including confidence intervals of the relative biases and error.

DWI model	Calibration parameters	Bias ⟨δ⟩ (rel. bias $\bar{\delta }$ [%])	Error ϵ (rel. error $\bar{\epsilon }$ [%])
SMI	{}	−0.17 (−39)	0.25 (55)
	$\left\{U_{2},s\right\}$	0.01 (1)	0.13 (29)
	$\left\{U_{2},s_{\text{pred}}\right\}$	−0.06 (−13)	0.16 (35)
BAYDIFF	{}	−0.33 (−73)	0.23 (50)
	$\left\{U_{2},s\right\}$	0.01 (2)	0.13 (29)
	$\left\{U_{2},s_{\text{pred}}\right\}$	−0.2 (−45)	0.29 (64)
WMTI–W^+^	{}	−0.26 (−58)	0.24 (53)
	$\left\{U_{2},s\right\}$	0.01 (1)	0.13 (28)
	$\left\{U_{2},s_{\text{pred}}\right\}$	−0.13 (−28)	0.21 (46)
WMTI	{}	−0.14 (−31)	0.21 (46)
	$\left\{U_{2},s\right\}$	0.0 (1)	0.12 (26)
	$\left\{U_{2},s_{\text{pred}}\right\}$	−0.04 (−9)	0.13 (29)
NODDI	{}	0.01 (3)	0.27 (59)
	$\left\{U_{2},s\right\}$	0.03 (6)	0.2 (44)
	$\left\{U_{2},s_{\text{pred}}\right\}$	0.07 (16)	0.18 (41)
NODDI–DTI	{}	−0.01 (−3)	0.26 (56)
	{s}	0.03 (7)	0.23 (51)
	$\left\{s_{\text{pred}}\right\}$	0.02 (5)	0.24 (52)

## 5 DISCUSSION

In this study, we demonstrated that the one‐to‐one correspondence between EM‐ and DWI‐based metrics of the axonal volume fraction could be improved by biophysically motivated, linear calibration with an offset associated with the volume fraction of unmyelinated axons and a scaling factor correcting for global compartmental $T_{2}$ relaxation differences. Using these calibration parameters, we found the best one‐to‐one correspondence between the EM‐based axon‐volume fraction and its WMTI‐based counterpart, closely followed by the SMI, BAYDIFF, and WMTI–W^+^ counterparts. Moreover, we proposed a method to predict the scaling parameter from known compartmental $T_{2}$ values. We showed that using a hybrid method that uses the predicted scaling parameter and only estimates the offset parameters achieved similar one‐to‐one correspondence between the EM‐based AVF and the DWI‐based counterparts when using the WMTI and SMI models.

### 5.1 Calibration parameters

The biophysical interpretation of the fitted calibration parameters potentially provides new insights into the DWI models investigated. Our hypothesis that the offset could be interpreted as a measure associated with the fraction of unmyelinated axons is supported by the observation that for all models with a high one‐to‐one correspondence the offset is particularly large for the Tsc2 mouse model and negligible for the other mouse models. This trend in the offset parameter follows the change in the fraction of unmyelinated axons between the Tsc2 mice and the other mouse models.

To estimate the fraction of unmyelinated axons per mouse model, we use the approximation that the total axonal volume fraction (i.e., the sum of myelinated and unmyelinated axons) is the same for all mouse models, although their relative proportion might change across mouse models. Given this approximation, the volume fraction of unmyelinated axons can be estimated as follows: observing from Figure 2 that the volume fraction of myelinated axons is about 0.35 and taking the percentage (of the fraction of all axons) of unmyelinated axons reported in the literature (e.g., 33% in Abdollahzadeh et al^25^ or 30% in Jelescu et al.^49^), the total axonal volume would be given approximately by ≈0.35/(1.0−0.33)≈0.52 (or 0.5 for Jelescu et al.). Assuming this total axonal volume fraction in Tsc2 mice to be the same as in control mice (i.e., 0.5), we can estimate from Figure 2 the volume fraction of unmyelinated axons to be 0.4 for Tsc2 mice.

Including an offset associated with the fraction of unmyelinated axons led to a substantial improvement in terms of ΔBIC for all models, except for the NODDI–DTI model. On the basis of the aforementioned simple approximation, we expect that the fraction of unmyelinated axons in Tsc2 mice is about 0.4, indicating that the offset $U_{2}$ in hypomyelinated mice of 0.18–0.24 (see Table 4) underestimates the volume fraction of unmyelinated axons.

Moreover, the fitted scaling parameter was smaller than its predicted counterpart for all two‐compartment models. For the four‐compartment NODDI model, however, the fitted scaling parameter was larger than its predicted counterpart. One reason for the mismatch between fitted and predicted scaling parameters might be that the proposed model for the predicted scaling parameter is not covering all the mechanisms that are driving the actual scaling process. Possible other factors could be, for example, nonmyelin macromolecules, which could also lead to an additional scaling effect ( $f_{A}=(1-f_{\text{NM}}-f_{M})f_{\text{AW}}$).^4^

Another study that compared WMTI with EM also estimated an offset and a scaling parameter.^49^ However, these results were not directly comparable with ours due to several experimental differences: (1) they compared in vivo DWI with ex vivo EM, whereas our DWI measurements were performed on ex vivo tissue samples, (2) in EM they assessed both unmyelinated and myelinated axons, while in our data only the myelinated axons were assessed, (3) they estimated the slope and offset for the axonal water fractions and not, as we did here, for the axonal volume fractions. Ignoring issue (1), one could convert their slope and offset into our parameters using the following equations: $s_{\text{pred}}^{\left(\text{Jelescu}\right)}=\left(1-f_{M}^{\left(\text{EM}\right)}\right)/slope$ and $U^{\left(\text{Jelescu}\right)}=offset\cdot \left(1-f_{M}^{\left(\text{EM}\right)}\right)/slope-f_{\text{A,U}}$. Here, we could not compare their slope and offset with ours because we did not have access to the individual myelin volume fractions $f_{M}^{\left(\text{EM}\right)}$ and the volume fractions of unmyelinated axons $f_{\text{A,U}}$ from Jelescu et al.^49^

### 5.2 Impact of calibration parameters on bias and error between EM and DWI

**FIGURE E1:**
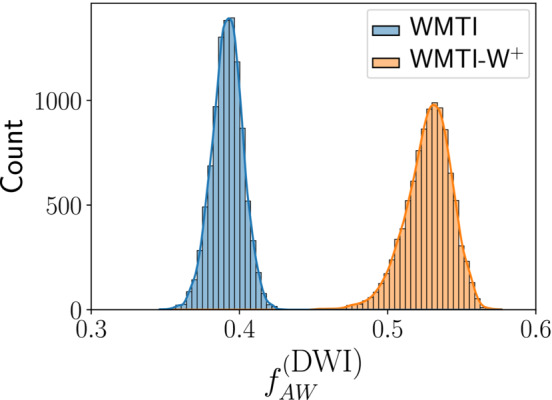
Histograms of results of axon water fraction estimated with WMTI–W^+^ (orange) and WMTI (blue) based on a noise simulation of 10 000 noise samples simulated for SNR=150.

All DWI models deviate clearly from the EM reference without additional calibration ( $\bar{\epsilon }\approx 46\%(\text{WMTI})-59\%(\text{NODDI})$, see Table 5). One reason for the observed difference is probably that the unmyelinated axons were not assessed with EM in this study, whereas the axonal water fraction measured with DWI is expected to be affected by both myelinated and unmyelinated axons. A comparison of the DWI‐based axon volume fraction with our EM data therefore requires accounting for this difference, which we sought to achieve through additional calibration parameters. While SMI benefits the most from calibration (by 26%), WMTI–W^+^, BAYDIFF, and WMTI followed closely (by 25%, 21%, and 20%, respectively), while calibration had much less of an impact on NODDI and NODDI–DTI (15% and 5%). In particular, DWI models with fewer free model parameters improved less through calibration. It is striking that relative bias and relative error were reduced most substantially for the DWI models that fitted the compartmental diffusivities (see Table 5). Although the relative errors of these DWI models are all within their mutual confidence intervals, as given in Table D1 in the Appendix, there is an apparent trend that WMTI is somewhat more accurate. In particular, that WMTI was slightly more accurate than WMTI–W^+^ was surprising, because WMTI–W^+^ also accounts for fibre dispersion whereas WMTI does not. This may be partly attributed to chance, but our observation that WMTI–W^+^ is more prone to noise (see simulations in Figure E1) is also in agreement with this trend. The larger number of free parameters in WMTI–W^+^ as compared with WMTI can lead to a less well‐conditioned optimisation prolem, which, in turn, could explain the higher noise susceptibility of WMTI–W^+^. Moreover, the corpus callosum and fornix, which were the focus of this study, have highly aligned fibres, making the neglect of fibre dispersion less relevant than in other areas of the brain with higher fibre dispersion.

### 5.3 Practical impact

The estimation of two calibration parameters, scaling and offset, is challenging, necessitating a well‐distributed set of data points with a wide dynamic range. Acquiring such a dataset presents a practical challenge, as achieving a wide dynamic range within the same tissue type (e.g., white matter) requires significant modulation of tissue composition. In our study, we addressed this challenge by utilizing a multimodal dataset,^38^ which included data from three mouse models with myelination ranging from hypo‐ to hypermyelinated, along with control mice.

However, applying this approach to humans is even more challenging, due to the limited availability of multimodal human data acquired with EM and DWI. To overcome this, we proposed a hybrid calibration approach that involves predicting a scaling factor and only fitting the remaining offset calibration parameter. We demonstrated that, for the standard and NODDI signal models with compartmental $T_{2}$ dependence (Equation 10), the scaling parameter for axonal volume calibration can be predicted independently of the MRI protocol using known compartmental $T_{2}$ values. Furthermore, for WMTI (and to a lesser degree for SMI) the hybrid calibration approach yielded results comparable with the control calibration method, where both calibration parameters were estimated. The practical relevance of the hybrid calibration method lies in the fact that, at least for the WMTI model, it can be nearly as accurate as the control calibration method, enabling more efficient estimation of the remaining offset calibration parameter. This will enable future studies to estimate the remaining offset calibration parameter from a limited amount of multimodal data, and is thus particularly relevant when used for human DWI applications where histological reference data are limited.

**FIGURE C1:**
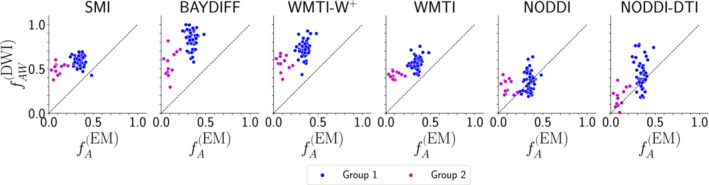
The scatter plots depict the compaprison of EM‐based axonal volume ( $f_{A}^{\left(\text{EM}\right)}$) and the axonal water fraction of the corresponding DWI models without rescaling by $1-f_{M}^{\left(\text{EM}\right)}$ as in Equation (3) (compare with Figure 4).

Overall, we found that the DWI model based axonal water fractions ( $f_{\text{AW}}^{\left(\text{DWI}\right)}$) (shown in Figure C1), and consequently also the axonal volume fractions $f_{A}^{\left(\text{DWI}\right)}$, are clearly less sensitive to demyelinating disease processes than a measure that is specifically assessing the fraction of myelinated axons. We believe that this is due to the sensitivity of the DWI‐based signal to both myelinated and unmyelinated axons. Thus, it might be relevant for the planning of future clinical studies investigating demyelination processes using MRI to complement the DWI with other imaging contrasts that are more specific to the myelin pool, for example, the magnetisation transfer saturation rate as obtained from multiparameter mapping^50^

### 5.4 Limitations

A number of limiting factors need to be considered when interpreting the results of our study. We made the strong but plausible assumption that the axon volume fraction across different mouse types is approximately constant. Since our reference EM measurements contained only the fraction of myelinated axons, we had to make this assumption to be able to test whether the offset is related to the fraction of unmyelinated axons. In principle, it is also possible to estimate the fraction of unmyelinated axons with EM.^25^, ^49^ However, those estimates have to be treated with caution because unmyelinated axons are more difficult to detect than myelinated axons even in high‐quality EM data. This is because unmyelinated axons have lower contrast in EM and are often smaller in size than myelinated axons, making it more challenging to estimate the fraction of unmyelinated axons accurately.^51^ This makes it likely that the estimated volume fraction of unmyelinated axons is less accurate than that of myelinated axons.

The assumption of no exchange between axonal and extracellular compartments implies that unmyelinated axons are impermeable, that is, the axonal cell membrane fully separates the intra‐ from the extra‐axonal water. Note that, even if the unmyelinated axons were partly permeable, the diffusion time used in the present study is short enough (12 ms) to justify the assumption of reduced exchange between intra‐ and extracellular water. In both cases, the fraction of unmyelinated axons would contribute to the DWI‐based axonal water fraction $f_{\text{AW}}^{\left(\text{DWI}\right)}$.

Furthermore, the assumption of highly aligned axons in WMTI might be violated because even the most aligned axons have an appreciable angular dispersion.^52^ However, it was proposed that WMTI can be used for voxels with an FA threshold larger than 0.4.^16^ This condition was violated in eight mouse–ROI combinations out of the total number of 60 combinations of our study. Despite these violations, the WMTI‐based $f_{A}^{\left(\text{DWI}\right)}$ showed the highest correspondence to its EM‐based counterpart $f_{A}^{\left(\text{EM}\right)}$.

We used in vivo $T_{2}$ values estimated across the entire human brain. However, compartmental relaxation times are likely to vary across fibre tracts, age, pathology, and between species. The proposed approach to estimate $s_{\text{pred}}$ is a first‐order approximation to correct for global compartmental $T_{2}$ differences. Despite these simplifications, the hybrid calibration approach using $s_{\text{pred}}$ worked almost as well for WMTI as the data‐driven calibration approach.

Of note was the fact that the remaining error was also rather large (26%) for the DWI models that fitted the compartmental diffusivities. This may be attributed partly to a potentially large variance in the reference, originating in the relatively small EM section size, which is probably not sufficient to capture the distribution of axons in the MRI voxels representatively. The cross‐sectional area of MRI voxels was ≈144 times larger for controls, and ≈187 for Pten, Rictor, and Tsc.

We estimated the theoretical scaling factors on the basis of in vivo compartmental $T_{2}$ values rescaled from 3T to 15.2T by a factor estimated from ex vivo values in human brain and finally compared them with ex vivo mouse models, which were thoroughly washed in PBS and Gd‐DTPA. At least for WMTI, the aforementioned limitations appear to be less relevant, since, for this DWI model, $\left\{U_{2},s_{\text{pred}}\right\}$ achieved similar accuracy to the combination $\left\{U_{2},s\right\}$.

Furthermore, our results are based on a multimodal dataset of fixed tissue acquired in three different mouse models. This might be a problem, since the fraction of unmyelinated axons might be different between humans and mice. However, it was shown that the fraction of unmyelinated axons is constant across species.^23^

A potential, unexplained factor affecting the calibration parameters in this study is represented by uncontrolled tissue deformations due to chemical and physical treatment of the tissue samples. Tissue shrinkage due to chemical fixation can be ruled out as a limiting factor for the comparability of DWI and EM data, since this study was performed on ex vivo DWI and EM data that underwent common chemical fixation procedures (see Section 3.1.1), However, preparation of the tissue samples for EM required additional steps such as dehydration in graded ethanol and the cutting of sections (Section 3.1.3). Dehydration in ethanol has been identified as a source for shrinkage, varying across whole‐brain samples between 2% and 3%.^53^ However, shrinkage at tissue surfaces can be larger. Shrinkage and expansion of tissue structures due to the cutting and unfolding of sections for EM also cannot be ruled out and their magnitude is difficult to assess. Visual inspection of the EM sections, however, suggested that such deformations were rather small.

Finally, some models account for the effect of fixation by incorporating an additional dot compartment. In our study, only the NODDI model accounted for the dot compartment explicitly. In vivo, it has been shown that the dot compartment can be neglected,^54^, ^55^ but it is debatable whether this applies to the ex vivo case as well.^19^ Although the other models neglected the dot compartment, the SMI, BAYDIFF, WMTI, and WMTI–W^+^ models described the EM data better when using calibration parameters.

The translation of our findings to the in vivo human situation might be confounded by the effect of the fixative on the DWI signal. Future studies assessing the effect of fixation on DWI data might help to translate the estimated calibration parameters into the in vivo situation.

### 5.5 Conclusion

In summary, we demonstrated that linear calibration with two biophysically motivated calibration parameters, an offset accounting for the volume fraction of unmyelinated axons and a scaling factor accounting for global compartmental $T_{2}$ differences, enhances agreement between EM‐ and DWI‐based estimates of the volume fraction of myelinated axons. Our findings suggest that, after calibration, the DWI models that fitted the compartmental diffusivities, that is, WMTI, BAYDIFF, WMTI–W^+^, and SMI, were the most acurate. Finally, we introduced a more efficient hybrid calibration approach, where only the offset is estimated but the scaling is predicted theoretically, and found that it was particularly accurate for WMTI, for which a similar one‐to‐one correspondence to EM was achieved using this approach. This makes the hybrid approach particularly appealing for usage in human brain data, where multimodal data are less common than for animals.

## Figures and Tables

**FIGURE C2:**
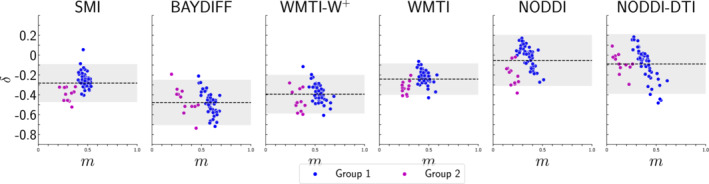
Bland–Altman plots of differences δ (Equation 15) versus mean m (Equation 16) between EM‐based axonal volume fraction ( $f_{A}^{\left(\text{EM}\right)}$) and DWI‐based axonal water fraction ( $f_{\text{AW}}^{\left(\text{DWI}\right)}$) corresponding to Figure 5 (individual data shown are in correspondence with Figure C1). The corresponding biases and errors are summarised in Table C1

## Data Availability

The data that support the findings of this study are available on request from the corresponding author. The data are not publicly available due to privacy or ethical restrictions.

## APPENDIX A ADDITIONAL DATA

### A.1

**TABLE A1:** Summary of all fitted models and calibration parameter combinations as shown in Figure 3. The BIC was determined on the basis of all available data. In contrast, $U_{1}$, $U_{2}$, and s were estimated in a leave‐one‐out fashion, in which each mouse individual was excluded from the computation once in order to get an estimate of the standard deviation (see also Table 4). The predicted scaling, $s_{\text{pred}}$, was calculated as described in Section 2.3.

DWI model	Calibr. parameters	BIC	$U_{1}$ ( SD)	$U_{2}$ ( SD)	s ( SD)	$s_{\text{pred}}$	Rel. Δs [%]
SMI	{}	−176	0 (–)	0 (–)	1 (–)	–	–
	$\left\{U_{1}\right\}$	−195	0.13 (0.0)	– (–)	1 (–)	–	–
	$\left\{U_{2}\right\}$	−230	– (–)	0.37 (0.01)	1 (–)	–	–
	$\left\{U_{1},U_{2}\right\}$	−292	0.13 (0.0)	0.37 (0.01)	1 (–)	–	–
	{s}	−250	– (–)	– (–)	0.62 (0.02)	0.89	−30.0
	$\left\{U_{1},s\right\}$	−246	0.0 (0.0)	– (–)	0.62 (0.02)	0.89	−30.0
	$\left\{U_{2},s\right\}$	−312	– (–)	0.24 (0.01)	0.72 (0.01)	0.89	−19.0
	$\left\{U_{1},U_{2},s\right\}$	−308	0.0 (0.0)	0.24 (0.01)	0.72 (0.01)	0.89	−19.0
BAYDIFF	{}	−116	0 (–)	0 (–)	1 (–)	–	–
	$\left\{U_{1}\right\}$	−172	0.31 (0.0)	– (–)	1 (–)	–	–
	$\left\{U_{2}\right\}$	−138	– (–)	0.44 (0.01)	1 (–)	–	–
	$\left\{U_{1},U_{2}\right\}$	−262	0.31 (0.0)	0.44 (0.01)	1 (–)	–	–
	{s}	−268	– (–)	– (–)	0.47 (0.01)	0.93	−49.0
	$\left\{U_{1},s\right\}$	−264	0.0 (0.0)	– (–)	0.47 (0.01)	0.93	−49.0
	$\left\{U_{2},s\right\}$	−313	– (–)	0.18 (0.0)	0.52 (0.0)	0.93	−44.0
	$\left\{U_{1},U_{2},s\right\}$	−309	0.0 (0.0)	0.18 (0.0)	0.52 (0.0)	0.93	−44.0
WMTI–W^+^	{}	−139	0 (–)	0 (–)	1 (–)	–	–
	$\left\{U_{1}\right\}$	−178	0.22 (0.0)	– (–)	1 (–)	–	–
	$\left\{U_{2}\right\}$	−173	– (–)	0.43 (0.0)	1 (–)	–	–
	$\left\{U_{1},U_{2}\right\}$	−275	0.22 (0.0)	0.43 (0.0)	1 (–)	–	–
	{s}	−258	– (–)	– (–)	0.52 (0.01)	0.89	−41.0
	$\left\{U_{1},s\right\}$	−254	0.0 (0.0)	– (–)	0.52 (0.01)	0.89	−41.0
	$\left\{U_{2},s\right\}$	−315	– (–)	0.21 (0.0)	0.59 (0.01)	0.89	−33.0
	$\left\{U_{1},U_{2},s\right\}$	−311	0.0 (0.0)	0.21 (0.0)	0.59 (0.01)	0.89	−33.0
WMTI	{}	−200	0 (–)	0 (–)	1 (–)	–	–
	$\left\{U_{1}\right\}$	−218	0.1 (0.0)	– (–)	1 (–)	–	–
	$\left\{U_{2}\right\}$	−255	– (–)	0.3 (0.0)	1 (–)	–	–
	$\left\{U_{1},U_{2}\right\}$	−311	0.1 (0.0)	0.3 (0.0)	1 (–)	–	–
	{s}	−264	– (–)	– (–)	0.68 (0.01)	0.89	−24.0
	$\left\{U_{1},s\right\}$	−260	0.0 (0.0)	– (–)	0.68 (0.01)	0.89	−24.0
	$\left\{U_{2},s\right\}$	−327	– (–)	0.21 (0.0)	0.76 (0.01)	0.89	−14.0
	$\left\{U_{1},U_{2},s\right\}$	−323	0.0 (0.0)	0.21 (0.0)	0.76 (0.01)	0.89	−14.0
NODDI	{}	−232	0 (–)	0 (–)	1 (–)	–	–
	$\left\{U_{1}\right\}$	−232	0.0 (0.0)	– (–)	1 (–)	–	–
	$\left\{U_{2}\right\}$	−259	– (–)	0.18 (0.01)	1 (–)	–	–
	$\left\{U_{1},U_{2}\right\}$	−255	0.0 (0.0)	0.18 (0.01)	1 (–)	–	–
	{s}	−232	– (–)	– (–)	0.97 (0.03)	0.9	7.0
	$\left\{U_{1},s\right\}$	−228	0.0 (0.0)	– (–)	0.97 (0.03)	0.9	7.0
	$\left\{U_{2},s\right\}$	−259	– (–)	0.21 (0.01)	1.11 (0.01)	0.9	22.0
	$\left\{U_{1},U_{2},s\right\}$	−255	0.0 (0.0)	0.21 (0.01)	1.11 (0.01)	0.9	22.0
NODDI–DTI	{}	−237	0 (–)	0 (–)	1 (–)	–	–
	$\left\{U_{1}\right\}$	−237	0.0 (0.0)	– (–)	1 (–)	–	–
	$\left\{U_{2}\right\}$	−240	– (–)	0.07 (0.0)	1 (–)	–	–
	$\left\{U_{1},U_{2}\right\}$	−236	0.0 (0.0)	0.07 (0.0)	1 (–)	–	–
	{s}	−244	0.0 (0.0)	0.0 (0.0)	0.87 (0.02)	0.89	−2.0
	$\left\{U_{1},s\right\}$	−240	0.0 (0.0)	– (–)	0.87 (0.02)	0.89	−2.0
	$\left\{U_{2},s\right\}$	−242	– (–)	0.05 (0.0)	0.88 (0.02)	0.89	−1.0
	$\left\{U_{1},U_{2},s\right\}$	−238	0.0 (0.0)	0.05 (0.0)	0.88 (0.02)	0.89	−1.0

## APPENDIX B ESTIMATION OF COMPARTMENTAL $T_{2}$ FROM THE LITERATURE

### B.1

For estimating $s_{\text{pred}}$, we converted the compartmental $T_{2}$ values $T_{\text{2, a}}(3T)\approx 83\;\text{ms}$ and $T_{\text{2, e}}(3T)\approx 59\;\text{ms}$ from Tax et al^55^ from 3T to 15.2T. The conversion factor r was estimated from average values for the transverse relaxation time in ex vivo human white matter on the basis of monoexponential models: $T_{2}(3T)=83.8\;\text{ms}$ ^56^ and $T_{2}(15.2T)\approx 33\;\text{ms}$ (from the supplementary material of West et al.^30^). The decrease of the relaxation time from 3T to 15.2T can then be estimated by the ratio

(B1)
$$r=T_{2}(15.2T)/T_{2}(3T)\approx 0.4.$$

With Equation (B1), the relaxation rates of the individual compartments can be estimated as

(B2)
$$\begin{array}{ll} T_{\text{2, a}}(15.2T) & =rT_{\text{2, a}}(3T)\approx 33\;\text{ms},\;\\ T_{\text{2, e}}(15.2T) & =rT_{\text{2, e}}(3T)\approx 23\;\text{ms}.\; \end{array}$$

Note that we assumed that the compartmental $T_{2}$ times in the fixed tissue were the same as for in vivo tissue. This assumption is based on the observation that washing the samples in PBS retrieves $T_{2}$ values similar to the in vivo case.^42^ From Equations (B2), the compartmental differences are then $\Delta_{e}\approx 12.6\;s^{-1}$ and $\Delta_{\text{iso}}\approx -32.0\;s^{-1}$, where $T_{\text{2,iso}}=1\;\text{ms}$ was assumed.^21^ For $T_{\text{2,dot}}$ no values could be found to the best of our knowledge. We assumed $T_{\text{2,dot}}=T_{\text{2,e}}$, that is, $\Delta_{\text{dot}}=\Delta_{e}$ in Equation (10). Estimates of the compartmental values were employed in the hybrid approach described in Section 3.4.3.

## APPENDIX C COMPARISON OF DWI‐BASED AXON WATER FRACTION AND EM‐BASED AXON VOLUME FRACTION

### C.1

In Figure C1 we compare the histological reference $f_{A}^{\left(\text{EM}\right)}$ with its DWI‐based counterpart $f_{\text{AW}}^{\left(\text{DWI}\right)}$. As previously reported, $f_{\text{AW}}^{\left(\text{DWI}\right)}$ correlates with $f_{A}^{\left(\text{EM}\right)}$ across all DWI models,^37^ but $f_{A}^{\left(\text{EM}\right)}$ is more sensitive to the demyelination process than $f_{\text{AW}}^{\left(\text{DWI}\right)}$ (see also the corresponding discussion in Section 5.3). Another interesting observation is that $f_{\text{AW}}^{\left(\text{DWI}\right)}$ from NODDI–DTI shows almost a one‐to‐one correspondence with $f_{A}^{\left(\text{EM}\right)}$. The corresponding Bland‐Altman plots are shown in Figure C2 and the relative biases and errors are summarised in Table C1.

**TABLE C1:** Bias (relative bias) and error (relative error) for the data points shown in Figure C2 (compare with Table 5). The definition of relative bias and error is given in Equations (18) and (19), respectively. Note that, for this table, $f_{A}^{\left(\text{DWI}\right)}$ was replaced by $f_{\text{AW}}^{\left(\text{DWI}\right)}$ in the equations.

DWI model	Bias ⟨δ⟩ (rel. bias $\bar{\delta }$ [%])	Error ϵ (rel. error $\bar{\epsilon }$ [%])
SMI	−0.28 (−62)	0.19 (42)
BAYDIFF	−0.39 (−87)	0.23 (51)
WMTI–W^+^	−0.48 (−106)	0.19 (43)
WMTI	−0.24 (−53)	0.16 (35)
NODDI	−0.05 (−12)	0.26 (57)
NODDI–DTI	−0.09 (−20)	0.3 (67)

## APPENDIX D CONFIDENCE INTERVALS FOR RELATIVE BIASES AND ERRORS

### D.1

In order to estimate confidence intervals for the relative biases and errors, we had to perform a slightly modified leave‐one‐out analysis compared with the one before (see also Table 5). There, the offsets $U_{j}$ and the scaling s were determined as averages over 15 samples each with one distinct mouse individual excluded. Then, biases and errors as given in Table 5 were determined. Here, we again computed offsets $U_{j}$ and scaling s 15 times each with one distinct mouse individual excluded, but now each of the 15 $U_{j}$ and s were used to compute 15 biases and errors in order to estimate their variances as given in Table D1. For this reason, the biases and errors in Tables 5 and D1 differ slightly.

**TABLE D1:** Table analogous to Table 5 but including confidence intervals ( ±1.96·SD) for the relative biases $\bar{\delta }$ and relative errors $\bar{\epsilon }$ estimated as described in Appendix D.

DWI model	Calibration parameters	Bias ⟨δ⟩ (rel. bias $\bar{\delta }\pm 1.96\cdot SD$ [%])	Error ϵ (rel. error $\bar{\epsilon }\pm 1.96\cdot SD$ [%])
SMI	{}	−0.17 (−39 ±5)	0.25 (56 ±5)
	$\left\{U_{2},s\right\}$	0.01 (1 ±1)	0.13 (29 ±1)
	$\left\{U_{2},s_{\text{pred}}\right\}$	−0.06 (−13 ±2)	0.16 (35 ±2)
BAYDIFF	{}	−0.33 (−74 ±7)	0.23 (51 ±4)
	$\left\{U_{2},s\right\}$	0.01 (2 ±1)	0.13 (30 ±2)
	$\left\{U_{2},s_{\text{pred}}\right\}$	−0.2 (−46 ±6)	0.29 (65 ±8)
WMTI–W^+^	{}	−0.26 (−59 ±6)	0.24 (54 ±6)
	$\left\{U_{2},s\right\}$	0.01 (1 ±1)	0.13 (29 ±3)
	$\left\{U_{2},s_{\text{pred}}\right\}$	−0.13 (−29 ±4)	0.21 (47 ±5)
WMTI	{}	−0.14 (−32 ±4)	0.21 (47 ±5)
	$\left\{U_{2},s\right\}$	0.0 (1 ±1)	0.12 (26 ±2)
	$\left\{U_{2},s_{\text{pred}}\right\}$	−0.04 (−9 ±1)	0.13 (29 ±3)
NODDI	{}	0.01 (3 ±3)	0.26 (60 ±6)
	$\left\{U_{2},s\right\}$	0.03 (6 ±1)	0.2 (45 ±4)
	$\left\{U_{2},s_{\text{pred}}\right\}$	0.07 (16 ±2)	0.18 (41 ±3)
NODDI–DTI	{}	−0.01 (−3 ±2)	0.26 (57 ±5)
	{s}	0.03 (7 ±2)	0.23 (52 ±5)
	$\left\{s_{\text{pred}}\right\}$	0.02 (5 ±2)	0.24 (53 ±5)

## APPENDIX E NOISE ANALYSIS OF WMTI AND WMTI–W^+^

### E.1

To test the hypothesis of whether WMTI–W^+^ is more prone to noise than WMTI, a noise simulation based on a variant of the standard model that uses the Watson distribution to model neurite dispersion (equation 1 in Jesperson et al.^36^) was performed. The signal model was used as a forward model and the integral was solved numerically (Matlab, Lebedev quadrature). First, noise‐free signals were simulated for the 61 gradient directions and b values that were used to acquire the mouse dataset. To simulate the noise‐free data, a set of biophysical parameters ( $\nu =0.73,\;K=8,\;D_{a}=2.0,\;D_{e}^{\|}=1.0,\;D_{e}^{⊥}=0.3$) describing highly aligned fibres was taken from Coelho et al.^57^ The noise‐free signals were then used to compute noisy magnitude signals according to $S_{\text{cont}}=|S_{\text{noisefree}}+a+b_{i}|$ (similar to Oeschger et al.^58^), where $a,b_{i}\in N(0,\sigma )$ were each randomly drawn from a zero‐mean Gaussian with standard deviation $\sigma =\sqrt{2}S_{0}/SNR$, where SNR is the signal‐to‐noise ratio and $S_{0}=1$. Following this procedure, 10 000 noise samples for SNR=150 were simulated and fitted with standard DKI (the NLLS algorithm from the ACID toolbox was used), the results of which could then be used to compute the axon water fraction $f_{\text{AW}}^{\left(\text{DWI}\right)}$ for WMTI–W^+^ and WMTI. Histograms of the results are shown in Figure E1. The histograms in Figure E1 were compared using the standard deviation, which served as a measure to quantify the spread of results of both methods induced by noise. The standard deviation of WMTI–W^+^ was 0.016, while the standard deviation of WMTI was 0.01. The fact that the standard deviation of WMTI–W^+^ was 60% larger than for WMTI supports the hypothesis that WMTI–W^+^ is more prone to noise than WMTI (see Section 5.2).

## APPENDIX F INSTRUCTIONS FOR DKI FITTING USING THE ACID TOOLBOX

### F.1

- Installation instructions for the ACID toolbox are described in detail here: https://bitbucket.org/siawoosh/acid-artefact-correction-in-diffusion-mri/wiki/Home
- In this article, we used this tagged version of the ACID toolbox: https://bitbucket.org/siawoosh/acid-artefact-correction-in-diffusion-mri/commits/d5ce665d709647aa1122c7b8b0b71420bc15e6e9
- To estimate DKI parameters we used the following batch: https://drive.google.com/file/d/1Iw-4EWk57IoMH5DWovzxz8Ru8f05U3-h/view?usp=drive_link. Please note that the corresponding paths might have to be adjusted to your local paths and that the data need to be requested from Mark D. Does.
